# Comparison of 4-chloro-2-nitrophenol adsorption on single-walled and multi-walled carbon nanotubes

**DOI:** 10.1186/1735-2746-9-5

**Published:** 2012-09-03

**Authors:** Ali Mehrizad, Mehran Aghaie, Parvin Gharbani, Siavoush Dastmalchi, Majid Monajjemi, Karim Zare

**Affiliations:** 1 Ph.D Student, Department of Chemistry, Science and Research Branch, Islamic Azad University, Tehran, Iran; 2 Faculty of Chemistry, North Tehran Branch, Islamic Azad University, Tehran, Iran; 3 Department of Chemistry, Ahar Branch, Islamic Azad University, Ahar, Iran; 4 Biotechnology Research Center, Tabriz University of Medical Sciences, Tabriz, Iran; 5 School of Pharmacy, Tabriz University of Medical Sciences, Tabriz, Iran; 6 Department of Chemistry, Science and Research Branch, Islamic Azad University, Tehran, Iran

**Keywords:** Carbon nanotube, 4-Chloro-2-nitrophenol, Adsorption, Isotherm model, Thermodynamic

## Abstract

The adsorption characteristics of 4-chloro-2-nitrophenol (4C2NP) onto single-walled and multi-walled carbon nanotubes (SWCNTs and MWCNTs) from aqueous solution were investigated with respect to the changes in the contact time, pH of solution, carbon nanotubes dosage and initial 4C2NP concentration. Experimental results showed that the adsorption efficiency of 4C2NP by carbon nanotubes (both of SWCNTs and MWCNTs) increased with increasing the initial 4C2NP concentration. The maximum adsorption took place in the pH range of 2–6. The linear correlation coefficients of different isotherm models were obtained. Results revealed that the Langmuir isotherm fitted the experimental data better than the others and based on the Langmuir model equation, maximum adsorption capacity of 4C2NP onto SWCNTs and MWCNTs were 1.44 and 4.42 mg/g, respectively. The observed changes in the standard Gibbs free energy, standard enthalpy and standard entropy showed that the adsorption of 4C2NP onto SWCNTs and MWCNTs is spontaneous and exothermic in the temperature range of 298–328 K.

## Introduction

Phenolic derivatives, such as a number of chloro- and nitrophenols are toxic and carcinogenic [1]. Chronic toxic effects due to phenols reported in humans include vomiting, difficulty in swallowing, anorexia, liver and kidney damage, headache, fainting and mental disturbances [2]. 4-Chloro-2-nitrophenol (4C2NP), which was chosen as the model compound in the present study, is recalcitrant and persistent towards biodegradation and is constituent intermediate of many industrial effluents [3]. While the World Health Organization (WHO) has recommended the permissible phenolic concentration of 0.001 mg/L in potable waters, the European Union (EU) has set a maximum concentration level of 0.5 μg/L of total phenols in drinking water [2,4]. Hence, removal of the phenolic compounds from wastewater before its discharge is necessary in order to reduce their side effects on the environment and human health.

Many methods such as biodegradation [5], photocatalytic degradation [6], ultrasonic degradation [7], solvent extraction [8], ozonation [9,10] and decomposition by Fenton reagents [11] may be used to remove phenolic materials from aqueous solution. However, by far, the most frequently used technology is adsorption by a solid phase.

Several different adsorbents such as activated carbon [12], silica [13], polymeric resins [14] and zeolites [15] have all been proposed to remove phenolic pollutants from wastewater. In most cases, the adsorbents have diameters in the range of submicron to micron and have large internal porosities to ensure adequate surface area for adsorption. However, the diffusion limitation within the particles leads to decreases in the adsorption rate and available capacity. Therefore, it is important and interesting to develop a novel adsorbent with a large surface area for adsorption, a small diffusion resistance and a high capacity.

The relative large specific surface area of carbon nanotubes (CNTs) enables them to become candidate for adsorption of phenolic compounds [16,17]. CNTs provide a large specific surface area and a strong Van der Waals binding energy for molecular adsorbates on well-defined adsorption sites such as interior, groove, exterior and interstitial sites [18].

The objective of the present work was to investigate the adsorption potential of single-walled and multi-walled carbon nanotubes (SWCNTs and MWCNTs) for removal of 4C2NP from aqueous solutions. Effects of contact time, CNTs dosage, pH and initial 4C2NP concentration on the adsorption capacity were studied. The Langmuir, Freundlich and Temkin isotherm models were used to describe the equilibrium data. The adsorption mechanisms of 4C2NP from aqueous solutions onto SWCNTs and MWCNTs were also evaluated in terms of thermodynamic parameters.

## Materials and methods

### Materials

Single-walled and multi-walled carbon nanotubes were purchased from Research Institute of Petroleum Industry (R.I.P.I), of Iran. On the basis of the information provided by the manufacturer, the CNTs were synthesized by catalytic chemical vapor deposition (CVD), and their characteristics are listed in Table 1. 4-Chloro-2-nitrophenol (C_6_H_4_ClNO_3_, M_w_ = 173.56 g/mol) was supplied by Fluka, Germany.

**Table 1:** Characteristics of SWCNTs and MWCNTs

**Adsorbent**	**Outer diameter (nm)**	**Length (μm)**	**Specific surface area (m**^**2**^**/g)**	**Purity (%)**
SWCNTs	1-2	5-15	550	>95%
MWCNTs	<10	5-15	280	>95%

### Adsorption experiments

Adsorption of 4C2NP onto CNTs was carried out by a batch method to obtain equilibrium data. The variation of 4C2NP concentration versus time in the aqueous solution was monitored under various conditions such as CNTs doses (0.005-0.5 g/250 mL), initial pH (2–12) and initial 4C2NP concentrations (2–10 mg/L). Stock solution was prepared by dissolving the required amount of 4C2NP in double distilled water (pH = 6). The initial pH was adjusted by adding either HCl or NaOH. Adsorption was achieved by adding a known amount of CNTs into 250 mL of 4C2NP solution of known concentration, pH and temperature in a 500 mL Erlenmeyer flask. The mixture was shaken in a shaking water bath at a speed of 140 shakes/min; samples were taken at predetermined time intervals and then centrifuged (Hettich/UNIVERSAL 16-R) at 10000 rpm for 10 min. Analysis of remained 4C2NP in the solution was done using a UV-160 Shimadzu UV–vis spectrophotometer at a wavelength of 219 nm (acidic pHs) or 234 nm (neutral and alkaline pHs). The adsorption yield (%) and amounts of adsorbed 4C2NP at equilibrium, i.e. *q*_*e*_ (mg/g), were calculated from the following equations (1–2), respectively:

(1) $$\text{Adsorption}\left(\%\right)=\left(\frac{C_{0}-C_{t}}{C_{0}}\right)\times 100$$

(2) $$q_{e}=\frac{\left(C_{0}-C_{e}\right)}{m}\times V$$

where C_0_, C_t_ and C_e_ are the initial, at any time t and equilibrium 4C2NP concentration (mg/L), respectively. V is the solution volume (L) and m is the adsorbent mass (g).

### Adsorption isotherm and thermodynamic studies

One of the most important studies to optimize the design of an adsorption process is to establish the adsorption isotherm. Adsorption isotherm studies were carried out with different initial 4C2NP concentrations ranging from 2 to 10 mg/L, with 0.1 g of CNTs at 25°C and pH = 6. Equilibrium adsorption isotherm data were analyzed according to the linear forms of Langmuir [19], Freundlich [20] and Temkin and Pyzhev [21] adsorption isotherm equations (35), respectively:

(3) $$\frac{1}{q_{e}}=\left(\frac{1}{K_{L}q_{m}}\right)\frac{1}{C_{e}}+\frac{1}{q_{m}}$$

(4) $$Lnq_{e}=\left(\frac{1}{n}\right)LnC_{e}+LnK_{F}$$

(5) $$q_{e}=B_{1}LnC_{e}+B_{1}LnK_{T}$$

Where, q_m_ is the maximum adsorption at monolayer (mg/g) and K_L_ is the Langmuir constant including the affinity of binding sites (L/mg). K_F_ and n are the Freundlich constants indicating adsorption capacity [(mg/g)(L/mg)^1/n^] and intensity, respectively. K_T_ and B_1_ are the Temkin constants (K_T_ is the equilibrium binding constant (L/g) and B_1_ is related to the heat of adsorption). The Langmuir isotherm can also be represented in terms of a dimensionless constant separation factor or an equilibrium parameter, R_L_, which is defined as:

(6) $$R_{L}=1/\left(1+K_{L}C_{0}\right)$$

The R_L_ value indicates the type of the isotherm to be either irreversible (R_L_ =0), favorable (0<R_L_ <1), linear (R_L_ =1) or unfavorable (R_L_ >1) [22].

Thermodynamic studies were performed to find the nature of adsorption process. These studies were carried out in the temperature range of 298–328 K, at an initial 4C2NP concentration of 10 mg/L, with 0.1 g of CNTs at pH = 6. Thermodynamic parameters, namely standard enthalpy (ΔH°), standard entropy (ΔS°) and Gibbs standard free energy (ΔG°) were calculated by using equations (79), [23]:

(7) $$LnK_{0}=-\frac{\Delta H^{ο}}{R}\left(\frac{1}{T}\right)+\frac{\Delta S^{ο}}{R}$$

(8) $$K_{0}=\frac{q_{e}}{C_{e}}$$

(9) $$\Delta G^{ο}=\Delta H^{ο}-T\Delta S^{ο}$$

where K_0_ is the equilibrium constant, T is the solution temperature (K) and R is the gas constant (8.314 J/mol.K) .

## Results

### Effect of contact time

**Figure 1:**
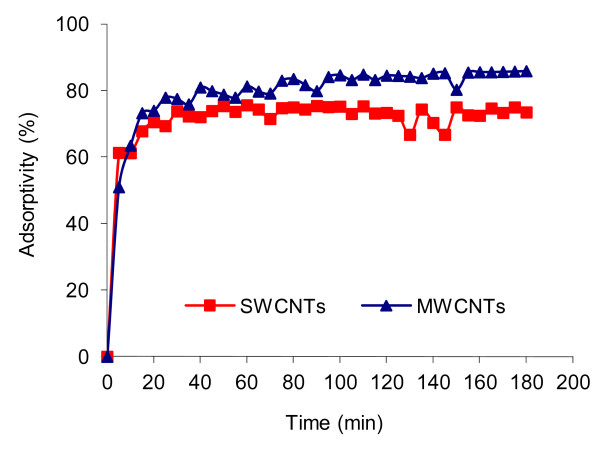
**Effect of contact time on 4C2NP adsorption onto CNTs; [4C2NP]**_**0**_ **= 10 mg/L; pH = 6; CNTs dose = 0.1 g; 25°C.**

In order to optimize contact time of adsorption, 0.1 g of adsorbent (SWCNTs or MWCNTs) was treated by 250 mL of 4C2NP solution with 10 mg/L concentration at 25°C. The working pH was that of stock solution (i.e., pH ≈ 6) and was not controlled. The mixture was shaken during different times and left for 180 min and then, was analysed (Figure 1). Rapid adsorption was observed during the first 20 min of contact time and no significant change in 4C2NP removal was observed after about 60 min. Therefore, 60 min was selected as the equilibrium time for both adsorbents in all experiments.

### Effect of CNTs dose

**Figure 2:**
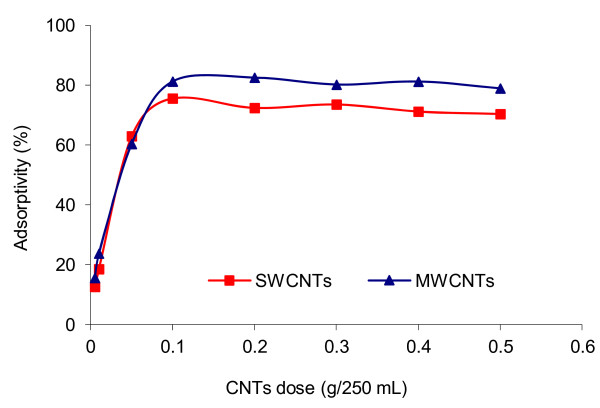
**Effect of CNTs dose on 4C2NP adsorption; [4C2NP]**_**0**_ **= 10 mg/L; pH = 6; 25°C; contact time = 60 min.**

To study the effect of SWCNTs and MWCNTs dose on the adsorption of 4C2NP, the experiments were done under the conditions described at previous stage with contact time of 60 min and variable CNTs dosage (0.005, 0.01, 0.05, 0.1, 0.2, 0.3, 0.4 and 0.5 g/250 mL). Figure 2 displays the effect of CNTs dose on the adsorption of 4C2NP. Along with the increase of adsorbent dosage (from 0.005-0.1 g), the percentage of 4C2NP adsorbed increased (from 12.64%-75.62% and 15.6%-81.28%) onto SWCNTs and MWCNTs, respectively. The adsorption of 4C2NP at adsorbent dosage higher than 0.1 g remained almost unchanged. As a result, the CNTs dose of 0.1 g was used in the subsequent experiments of this work.

### Effect of initial pH

**Figure 3:**
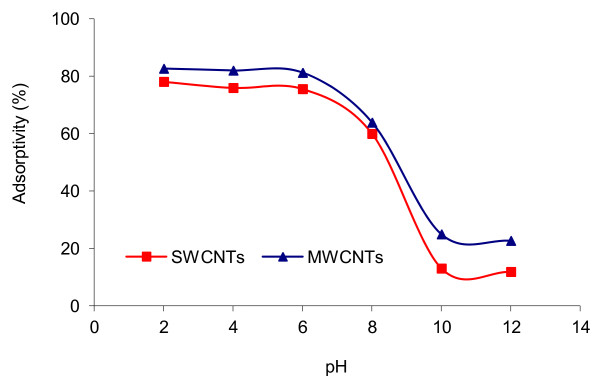
**Effect of pH on 4C2NP adsorption onto CNTs; [4C2NP]**_**0**_ **= 10 mg/L; CNTs dose = 0.1 g; 25°C; contact time = 60 min.**

In Figure 3, the effect of initial pH on the adsorption of 4C2NP onto SWCNTs and MWCNTs at 25°C and initial 4C2NP concentration of 10 mg/L is depicted. As shown, adsorption of 4C2NP remained almost unchanged up to pH = 6, but dropped drastically for pH >6. Thus in the rest of the experiments, to achieve the appropriate condition of adsorption, the pH was that of stock solution (pH ≈ 6) and was not controlled.

### Effect of initial concentration

**Figure 4:**
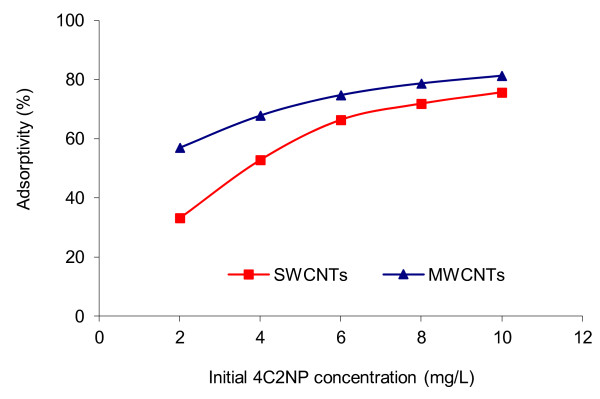
Effect of 4C2NP initial conc. on its adsorption onto CNTs; pH = 6; CNTs dose = 0.1 g; 25°C; contact time = 60 min.

The adsorption of 4C2NP was carried out at different initial concentrations ranging from 2 to 10 mg/L onto SWCNTs and MWCNTs at 25°C. As shown in Figure 4, when the initial concentration increased from 2 to 10 mg/L, the adsorption increased from 33.19% to 75.62% and from 56.96 to 81.28% onto SWCNTs and MWCNTs, respectively.

In order to compare the results of adsorption of 4C2NP onto SWCNTs and MWCNTs at various conditions such as pH, CNTs dosage and initial 4C2NP concentration, the difference of 4C2NP adsorption by SWCNTs and MWCNTs has been evaluated by *t-test* and are recorded in Table 2. As *t*_*critical*_*>t*_*sample*_, there was no significant difference between the adsorption of 4C2NP onto SWCNTs and MWCNTs (p = 0.05).

**Table 2:** Results of *t-test*

**Parameter**	***t-test***
pH	0.410
CNTs dosage	0.432
Initial 4C2NP concentration	1.344

### Adsorption isotherm studies

The values of Langmuir, Freundlich and Temkin parameters were calculated from the slope and intercept of linear plots of 1/q_e_ vs. 1/C_e_, Lnq_e_ vs. LnC_e_ and q_e_ vs. LnC_e_ (equations 3–5) respectively. The isotherm constants along with the correlation coefficients are listed in Table 3. It can be seen from Table 3, that the Langmuir isotherm better fited the experimental data (R^2^ >0.99) than the other isotherms.

**Table 3:** Isotherm parameters for 4C2NP adsorption onto CNTs

**Adsorbent**	**C**_**0**_	**Langmuir**			**Freundlich**			**Temkin**			
		**q**_**m**_	**K**_**L**_	**R**_**L**_	**R**^**2**^	**n**	**K**_**F**_	**R**^**2**^	**B**_**1**_	**K**_**T**_	**R**^**2**^
SWCNTs	2	1.444	0.406	0.55	0.993	0.242	2.088	0.901	27.434	0.3	0.854
	4			0.38							
	6			0.29							
	8			0.23							
	10			0.19							
MWCNTs	2	4.426	0.459	0.52	0.994	0.393	3.962	0.893	21.371	1.217	0.884
	4			0.35							
	6			0.26							
	8			0.21							
	10			0.17							

### Adsorption thermodynamic studies

**Figure 5:**
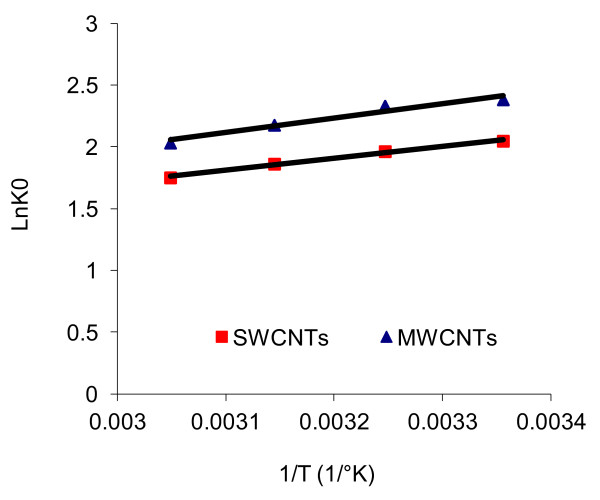
**Plot of LnK**_**0**_**vs. 1/T; [4C2NP]**_**0**_ **= 10 mg/L; pH = 6; CNTs dose = 0.1 g; contact time = 60 min.**

ΔH° and ΔS° were calculated from the slope and intercept of the plot of LnK_0_ vs. 1/T using the equation 7 (Figure 5). Standard Gibbs free energies at different temperatures were calculated from equation 9 and the results are summarized in Table 4.

**Table 4:** Thermodynamic parameters for 4C2NP adsorption onto CNTs

**ΔG° (kJ/mol)**					**ΔH° (kJ/mol)**	**ΔS° (J/mol.K)**	**R**^**2**^
	298 K	308 K	318 K	328 K			
SWCNTs	−5.100	−5.001	−4.902	−4.804	−8.043	−9.875	0.991
MWCNTs	−5.985	−5.858	−5.731	−5.604	−9.769	−12.697	0.954

## Discussion

As shown in Figure 1, a two-stage kinetic behavior was observed: rapid initial adsorption in a contact time of 20 min, followed by a second stage with a much lower adsorption rate. This is obvious from the fact that a large number of vacant surface sites are available for the adsorption during the initial stage and with the passage of time, the remaining vacant surface sites are difficult to be occupied due to repulsive forces between the solute molecules on the solid phase and in the bulk liquid phase [24]. Also Figure 1, shows that MWCNTs had slightly higher adsorbability than SWCNTs, despite the fact that SWCNTs have a higher specific surface area (550 m^2^/g) than MWCNTs (280 m^2^/g). This observation is similar with other researches [17], which may be explained by the presence more active adsorbing sites for MWCNTs compared SWCNTs. While MWCNTs have three possible sites for adsorption including external surface, inner cavity (the spaces in the innermost graphite tube) and interwall spaces (the spaces between the coaxial tubes of MWCNTs), SWCNTs have only two possible sites including external surface and inner cavity [18].

Based on the results (Figure 2), an increase in the adsorption with the increase of adsorbent dosage from 0.005-0.1 g can be attributed to greater surface area and the availability of more adsorption sites. Above 0.1 g of adsorbent dosage, the adsorption equilibrium of 4C2NP was reached and the removal of 4C2NP remained almost constant.

The solution pH is one of the key factors that controls the adsorption process on carbon materials; it controls the electrostatic interactions between the adsorbent and the adsorbate [24]. The pH of the zero point of charge (pH_zpc_) for SWCNTs and MWCNTs are 2.2 and 4.7-6.4, respectively [25]. At pH <6, since there is no electrostatic repulsion between the non-ionized 4C2NP species and the CNTs surfaces, the adsorption is higher. On the other hand for pH >pK_a_ (4-chloro-2-nitrophenol, pK_a_ = 6.46, 25°C), the phenols dissociate, forming phenolate anions, while the surface of CNTs is negatively charged. The electrostatic repulsion between the identical charges lowers the adsorption capacities. Besides, the phenolate anions are more soluble in the aqueous solution, and stronger adsorbate-water bonds must be broken before adsorption can take place [26]. Similar results were reported for the adsorption of phenol and cresol on carbon nanoporous [24] and 2-nitrophenol by MWCNTs [16].

It is clear from Figure 4, that the adsorption of 4C2NP onto CNTs was increased by increasing the initial 4C2NP concentration. These results may be explained by the fact that, the initial concentration provides the necessary driving force to overcome the resistances to the mass transfer of 4C2NP between the aqueous and the solid phases. Furthermore, the increase of loading capacity of CNTs with increasing initial 4C2NP concentration may be due to higher interaction between 4C2NP and CNTs [27].

Table 3 revealed that the adsorption of 4C2NP onto CNTs can be described with Langmuir isotherm. It can be seen from this Table that the calculated *R*_*L*_ values were between 0 and 1 for both the adsorbents, indicating favorable conditions for the adsorption process. *K*_*L*_ values for adsorbents followed the order of MWCNTs >SWCNTs, suggesting that the affinity of the binding sites for 4C2NP on CNTs also followed this order.

According to the results (Table 4), the negative values of Δ*G°* show a spontaneous nature of adsorption. The decrease in ΔG° with the decrease of temperature reveals more efficient adsorption at lower temperatures. In addition, a comparison of the standard Gibbs free energy values indicates that the adsorption onto MWCNTs is more spontaneous than SWCNTs. Hence, the amounts of ΔG° imply that the adsorption affinity of 4C2NP onto MWCNTs is stronger than SWCNTs. The negative value of ΔH° suggests that the 4C2NP-CNTs interaction is exothermic, which is the reason for the increase in adsorption at lower temperatures. Also, it suggests that the rise in the solution temperature dose not favor 4C2NP adsorption on either SWCNTs or MWCNTs. The same effect was reported for pentachlorophenol on MWCNTs [17]. The standard entropy change was found to have negative values for those processes. It mirrors a decrease in the randomness at solid-solution interface during the adsorption of 4C2NP onto SWCNTs and MWCNTs surfaces.

## Competing interests

The authors declare that they have no competing interests.

## Authors’ contributions

AM and PG carried out the isotherms studies and participated in the drafted the manuscript. MA and SD carried out the effect of different parameters studies. MM and KZ carried out the thermodynamic Studies. All authors read and approved the final manuscript.
